# Deep sequencing of the X chromosome reveals the proliferation history of colorectal adenomas

**DOI:** 10.1186/s13059-014-0437-8

**Published:** 2014-08-30

**Authors:** Anna De Grassi, Fabio Iannelli, Matteo Cereda, Sara Volorio, Valentina Melocchi, Alessandra Viel, Gianluca Basso, Luigi Laghi, Michele Caselle, Francesca D Ciccarelli

**Affiliations:** Department of Experimental Oncology, European Institute of Oncology (IEO), Milan, 20139 Italy; Division of Cancer Studies, King’s College London, London, SE1 1UL UK; Cogentech-IFOM Istituto FIRC di Oncologia Molecolare, Milan, 20139 Italy; CRO Aviano National Cancer Institute, Aviano, (PN) 33081 Italy; Laboratory of Molecular Gastroenterology, Department of Gastroenterology, Humanitas Clinical and Research Center, Via Manzoni 56, Rozzano (MI), 20089 Italy; Department of Theoretical Physics and INFN University of Turin, Turin, 10125 Italy; Present address: Department of Biosciences, Biotechnology and Biopharmaceutics, University of Bari, Bari, 70125 Italy

**Keywords:** Colorectal cancer, Cancer genomics, Clonal evolution, Tumor proliferation history

## Abstract

**Background:**

Mismatch repair deficient colorectal adenomas are composed of transformed cells that descend from a common founder and progressively accumulate genomic alterations. The proliferation history of these tumors is still largely unknown. Here we present a novel approach to rebuild the proliferation trees that recapitulate the history of individual colorectal adenomas by mapping the progressive acquisition of somatic point mutations during tumor growth.

**Results:**

Using our approach, we called high and low frequency mutations acquired in the X chromosome of four mismatch repair deficient colorectal adenomas deriving from male individuals. We clustered these mutations according to their frequencies and rebuilt the proliferation trees directly from the mutation clusters using a recursive algorithm. The trees of all four lesions were formed of a dominant subclone that co-existed with other genetically heterogeneous subpopulations of cells. However, despite this similar hierarchical organization, the growth dynamics varied among and within tumors, likely depending on a combination of tumor-specific genetic and environmental factors.

**Conclusions:**

Our study provides insights into the biological properties of individual mismatch repair deficient colorectal adenomas that may influence their growth and also the response to therapy. Extended to other solid tumors, our novel approach could inform on the mechanisms of cancer progression and on the best treatment choice.

**Electronic supplementary material:**

The online version of this article (doi:10.1186/s13059-014-0437-8) contains supplementary material, which is available to authorized users.

## Background

**Figure 1:**
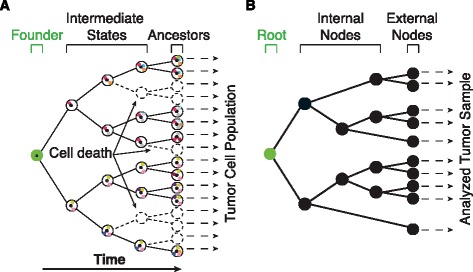
**Evolutionary model of tumor clonal expansion. (A)** Expansion of a monoclonal adenoma represented as a rooted binary tree [2]. Colored dots indicate somatic mutations that progressively occur during tumor development and are inherited by the surviving progeny. **(B)** Tree recapitulating the proliferation as inferred from the mutation profile. The combination of nodes in the tree reveals the occurrence of selection and cell death during tumor proliferation.

According to the model of clonal evolution, a solid and monoclonal tumor develops from a single mutated cell that progressively forms a mass of genetically heterogeneous cancer cells [1]. The initial phases of tumor expansion can be represented as a rooted binary tree [2] where daughter cells inherit all mutations of the parent, acquire new ones, and pass old and new mutations to the progeny (Figure 1A). The genomic modifications detectable in the final population are those inherited in the cell lineages that survived extinction, and the frequency of each mutation depends on the fraction of cells bearing it. Clonal mutations have the highest frequency because they were present in the founder cell at the root of the tree and are then inherited in all tumor cells. Subclonal mutations were instead acquired during the formation of the tumor mass. In case of neutral evolution, the frequency of subclonal mutations depends on the time when they were acquired: the earlier the acquisition, the higher the frequency. In case of mutations occurring in cells under selection, instead, the frequency does not directly reflect the acquisition time and mutations occurred later can have high frequency. In either case, the frequency of each somatic mutation is proportional to the fraction of mutated cells. Thus, the tumor mutation profile, that is, the collection of clonal and subclonal mutations, harbors the relics of the tumor evolutionary history and can be used to reconstruct the tumor proliferation tree (Figure 1B). Keys for a reliable tree reconstruction are a sensitive detection of subclonal mutations and a reliable estimation of their frequency. A few labs including ours have shown that next generation sequencing (NGS) can be readily used to identify subclonal mutations [3-5]. Sensitivity of NGS in detecting rare mutations increases with the depth of coverage, that is, with the number of times a given nucleotide position is sequenced. This property of NGS has been applied to rebuild tumor evolution through the identification of alterations occurring in the genome of single cancer cells [6-8] or in the whole cancer cell population [9-14]. These studies aimed to investigate genetic heterogeneity within primary tumors [7,8,11], and between primary tumors and metastases [6,9,10]. More recently, the mutation clonal-subclonal hierarchy has been used to rebuild carcinogenesis in prostate cancer [12] and multiple myeloma [13].

Here, we rebuilt the early phases of tumor development in four mismatch repair (MMR) deficient colorectal adenomas using the mutation profiles of the corresponding cancer cell population. We focused our analysis on colorectal adenomas because these tumors progress through a well-defined multistep genetic and histopathological succession of events [15]. Moreover, although there are a few reports of a possible polyclonal origin of polyps (see for example [16]), colorectal adenomas are thought to descend from one cell of origin located at the basis of the colon crypt [17-20]. Finally, owing to defects to the mismatch repair system, these tumors have high mutation rate owing to the mutator phenotype, but low chromosomal instability [21-23]. To infer the mutation profiles of these tumors, we deep sequenced the X chromosome of male patients. The high depth of coverage and the presence of only one copy of the X chromosome allowed the identification of subclonal mutations with a precise estimate of their frequency. We developed a novel approach to call high and low frequency mutations, to cluster them according to their frequencies and to finally rebuild the proliferation trees directly from these mutation clusters. The trees from the four patients recapitulated the evolutionary history of the original tumor and allowed to estimate the dynamics of their growth. We propose that similar analyses may be extended to other solid tumors to better understand the mechanisms of their development and to suggest the most effective therapeutic approach.

## Results

### Mutation profiles of the X chromosome in four MMR-deficient colorectal adenomas

**Figure 2:**
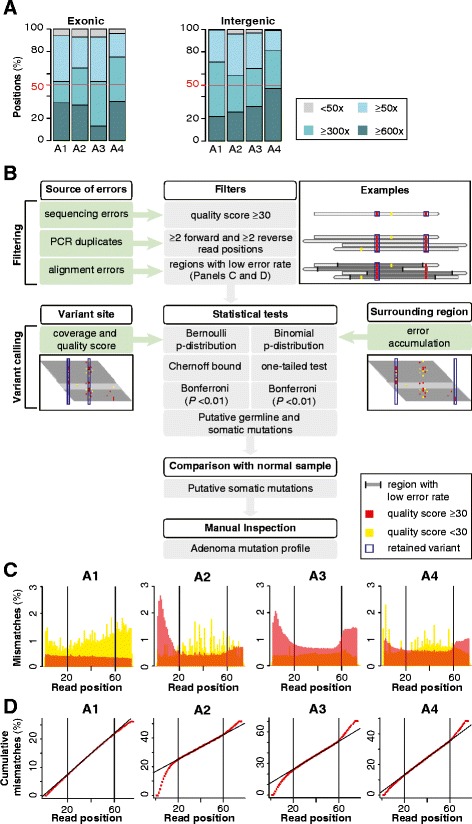
**Sequencing throughput and strategy for variant calling. (A)** Uniformity of coverage in the exonic and intergenic targeted regions. The mean coverage in each sample is highlighted in red. More than 50% of targeted regions were sequenced at least at 300× coverage in all four tumors. **(B)** Pipeline for variant calling. First, filters for quality scores and for propensity to accumulate errors were applied. Second, statistical tests were applied to account for the coverage and quality score of the variant site (Bernoulli distribution and Chernoff bound) and for the error accumulation of the surrounding region (Binomial distribution). Each test was performed on forward and reverse reads independently, and the resulting four *P*s were adjusted using Bonferroni correction. Candidate variants were retained if they passed all filters on mismatches and all statistical tests of the variant calling. The resulting ensemble of all somatic mutations at various frequencies constituted the adenoma mutation profile. **(C)** Variation of the quality score at different positions along the read. In sample A1 mismatches were evenly distributed along the read, with a slight decrease towards the end. In the other samples there was higher occurrence of mismatches at the beginning and at the end of the read, indicating that these positions were prone to accumulate errors. **(D)** Cumulative percentage of mismatches at each read position for base calls with quality score ≥30. In each sample, we only considered the portion of the reads where a linear correlation was observed. This corresponded to positions (20 to 76) for sample A1 and to positions (20 to 60) for samples and A2, A3, A4.

**Figure 3:**
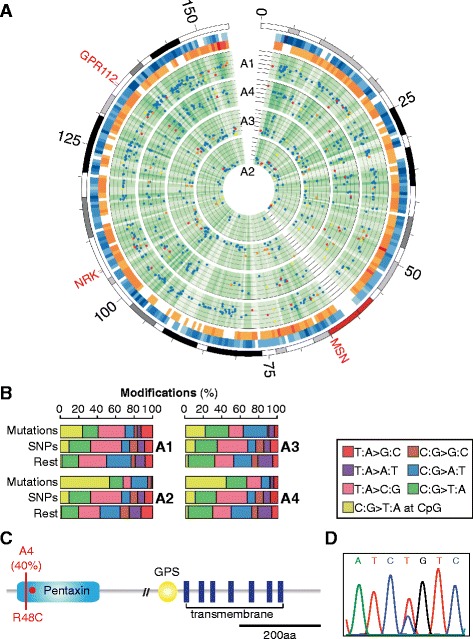
**Mutation profiles of the four adenomas. (A)** From the outer to the inner circles, plots display the entire X chromosome, the intergenic (blue, approximately 15 Mbp) and genic (orange, approximately 2 Mbp) targeted sites, and the sequenced sites (green) in the four samples. Genomic regions were divided into 500 kbp bins and color gradient represents target (blue and orange) and coverage (green) density. The six concentric circles in each sample represent decreasing mutation frequencies, from 60% to 10%. Dots depict intergenic (blue), non-coding (yellow), synonymous (orange), and non-synonymous (red) somatic mutations. The three cancer genes [27-29] with non-synonymous mutations are also highlighted. **(B)** Mutation pattern of somatic mutations, SNPs, and the rest of mismatches in the four samples. The mutation profile of each adenoma shows the typical pattern of MMR-deficient colorectal tumors [30]. **(C)** Schematic representation of the functional domains of the GPR112 protein, with the non-synonymous mutation in sample A4 (red line) and the missense mutation previously reported in colorectal cancer (CRC) (R54X, red circle) [28]. **(D)** Electropherogram of the R48C mutation occurring in the pentaxin domain of GPR112 in sample A4.

We captured all protein-coding exons and selected intergenic regions of the X chromosome for a total of more than 17 Mbp of DNA from four MMR-deficient adenomas and matching normal counterparts deriving from four distinct male patients (samples A1, A2, A3, A4, Table 1). Using Illumina deep sequencing, we produced a total amount of more than 3 billion raw reads. After alignment to the reference human genome and in accordance to literature [24,25], around 50% of the raw reads (corresponding to around 60% of all aligned reads) were mapped on target and the mean depth of coverage was higher than 300× in all samples (Figure 2A). In order to identify subclonal mutations at low frequency, we developed a novel analytical pipeline able to detect mutations and exclude possible errors. The pipeline was composed of initial filtering steps to remove PCR duplicates, sequencing, and alignment errors that have a sample-specific occurrence likely due to the sequencing performances (Figure 2B). In particular, we noticed that in the frozen sample A1 mismatches with good quality score (Q ≥30) were overall evenly distributed with a slight decrease toward the end of the reads, while in the three FFPE samples there was an accumulation of mismatches at the beginning and at the end of the reads (Figure 2C). For this reason, in each of the four samples, we only retained positions with a uniform mismatch distribution along the read (Figure 2D). We then performed the proper variant calling only on the retained positions. We took into account the coverage and the quality score of each variant site using the Bernoulli distribution and the Chernoff bound, and measured the propensity of the surrounding regions to accumulate errors with the binomial test (Figure 2B and Methods). We adjusted for multiple comparisons using Bonferroni correction and only mutations that passed all tests were further retained. Germline mutations were identified and discarded after comparison with the normal counterpart of each adenoma. All remaining mutations underwent visual inspection and the final pool of clonal and subclonal mutations constituted the mutation profile of the tumor (Figure 3A, Table 2, and Table S1 in Additional file 1). For each sample, we validated representative mutations and assessed overall high accuracy, ranging from 85% to 100% (Table 3 and Figure S1 in Additional file 2). We also detected the same low frequency mutations in multiple paraffin sections of the same tumor, thus confirming the monoclonal origin of the analyzed cancer cell populations.

**Table 1:** **Sample description**

**Sample**	**Age (years)**	**Germline mutation**	**Tumor histology**	**MMR immunohistochemistry**	**MSI**		**BRAF somatic mutations** ^**b**^	**KRAS somatic mutations** ^**c**^	**Other somatic modifications**
					**Unstable/Total**	**Markers**			
A1	44	MSH2: c.(?_-68)_792 + ?del (deletion of exons 1 to 4)	Low-grade tubular adenoma with focal high-grade dysplasia, ≥50% tumor content^a^	Loss of MSH2	5	BAT25, BAT26, NR21, NR24, MONO-27	Wild type	Wild type	-
A2	50	MSH2: c.2738delC	Flat adenoma with low to high dysplasia	Loss of MSH2	3	BAT26 (minor fraction), NR21, MONO-27	Wild type	Wild type	-
A3	77	-	85% well differentiated, early adenocarcinoma with up to severe dysplasia	Loss of MLH1	4	BAT26, BAT25, NR24, MONO-27	V600E	Wild type	*MLH1* hypermethylation
A4	48	MSH2: c.1216C > T	60% adenomatous tissue, 25% well-differentiated adenocarcinoma extending into the muscular layer	Loss of MSH2	4	BAT26, NR21, NR24, MONO-27	Wild type	Wild type	-

For each sample, age at the time of tumor resection, germline mutation, tumor histology, immunohistochemistry of mismatch repair (MMR) proteins, measure of microsatellite instability (MSI), and somatic mutation in colorectal cancer hotspots are reported. Germline mutations are described according to the Human Genome Variation Society (http://www.hgvs.org/mutnomen). MSI was assessed by checking for a panel of five unstable microsatellite markers (BAT25, BAT26, NR21, NR24, and MONO-27).

^a^Tumor content was inferred from the MSI spectrum [26].

^b^Mutation V600E was screened by TaqMan assay in samples A2, A3, A4 and by direct sequencing of *BRAF* exon 15 in sample A1.

^c^ *KRAS* exons 2 and 3 were sequenced in all samples, except for sample A1 where only exon 2 was screened.

**Table 2:** **Mutation profiles and indels of the four adenomas**

**Sample**	**Filtering steps**	**Variant calling**	**SNPs**	**Somatic mutations** ^**a**^	**Gold set** ^**b**^	**Mutations/Mbp** ^**c**^	**Indels** ^**d**^
A1	9,717	9,021	8,586	367	359	21	80
A2	7,681	7,194	7,095	56	37	3	2
A3	10,632	8,704	7,328	168	87	10	20
A4	10,594	8,826	8,516	180	172	11	31

^a^Mutations retained after manual inspection.

^b^Mutations with frequency ≥4% and occurring in >6 different read positions.

^c^Mbp on target are 17.2, 16.7, 16.5, and 17.1 for A1, A2, A3, and A4, respectively.

^d^Only high frequency indels are shown (frequency ≥20%). Overall, only three indels introduced frameshifts and likely activated the non-sense mediated decay (Table S2 in Additional file 3). These modifications were not considered for clustering and tree reconstruction because their frequency cannot be precisely measured.

**Table 3:** **Accuracy of variant calling**

**Sample**	**All mutations**			**4% ≤ Frequency <10%**		
	**Tested**	**Validated**	**Accuracy**	**Tested**	**Validated**	**Accuracy**
	**(% total)**			**(% total)**		
A1	24 (7%)	24	100%	4 (25%)	4	100%
A2	11 (20%)	9	82%	2 (9%)	2	100%
A3	20 (12%)	17	85%	4 (6%)	2	50%
A4	14 (8%)	13	93%	3 (14%)	3	100%
Overall	69 (9%)	63	91%	13 (10%)	11	85%

Mutations were randomly selected and tested with either Sanger sequencing (frequency ≥10%) or TaqMan assay (4% ≤ frequency <10%). TaqMan results for mutations with frequency <4% were inconclusive, likely due to the detection limit of the assay (see Table S1 in Additional file 1). Accuracy was estimated as the fraction of confirmed variants over the total pool of tested variants.

In all samples, the signature of somatic mutations showed the typical pattern of MMR-deficient tumors [23,30], with a prevalence of C:G to T:A transversions when compared to SNPs and to the rest of mismatches (Figure 3B). Interestingly, the number of somatic variants of the four mutation profiles was in agreement with the degree of genomic instability as inferred from the MSI spectrum (Tables 1 and 2). In particular, sample A1, which was a highly unstable adenoma, also showed the highest number of mutations. On the contrary, the mutation frequency of sample A2 was of around three mutations per mega base pairs, which is in the range of mutation frequency of MSI low CRCs [23]. We also reassessed the MMR status of sample A2 and confirmed the loss of MSH2 and the low levels of MSI (Figure S2 in Additional file 2). These observations indicate that our strategy succeeded in recapitulating the mutation landscape of each tumor both qualitatively and quantitatively. In all samples, the vast majority of the detected mutations (638 out of 771 total mutations, Table S1 in Additional file 1) fell in intergenic regions, while 49 out of the 133 mutations that hit protein-coding exons also led to amino acid changes. Three of these non-synonymous mutations modified known cancer genes (*MSN*, *NRK*, *GPR112*) [27-29,31]. Interestingly, *GPR112* has been already reported as a potential driver gene in CRC [28] (Figure 3C and D).

In addition to single nucleotide variants, we also identified high frequency small insertions and deletions (indels, Table 2). Again in line with what has been previously reported [30], we observed a lower occurrence of indels in comparison to single nucleotide modifications. Four of these indels occurred in coding exons and led to the frameshift of the corresponding codon (Table S2 in Additional file 3). Owing to the difficulty of correctly assessing their frequency, indels were not used for rebuilding the proliferation trees.

In addition to the whole set of single nucleotide variants, in each tumor we identified a gold set of highly reliable mutations with frequency ≥4% and supported by more than six reads starting at different genomic positions (Table S1 in Additional file 1). The lower bound of frequency was set because we could not determine whether mutations with frequency <4% were true or not, due to sensitivity limits of the TaqMan assay that we used for the orthogonal validation. The support of more than six reads starting at different positions excluded variants that, although true, could have inaccurate frequency estimation due to PCR amplification (Figure S1 in Additional file 2). As described below, in each tumor we used the gold set as a control and rebuilt in parallel two independent tumor proliferation trees using the whole and the gold sets of mutations.

### Mutation clusters from mutation profiles

**Figure 4:**
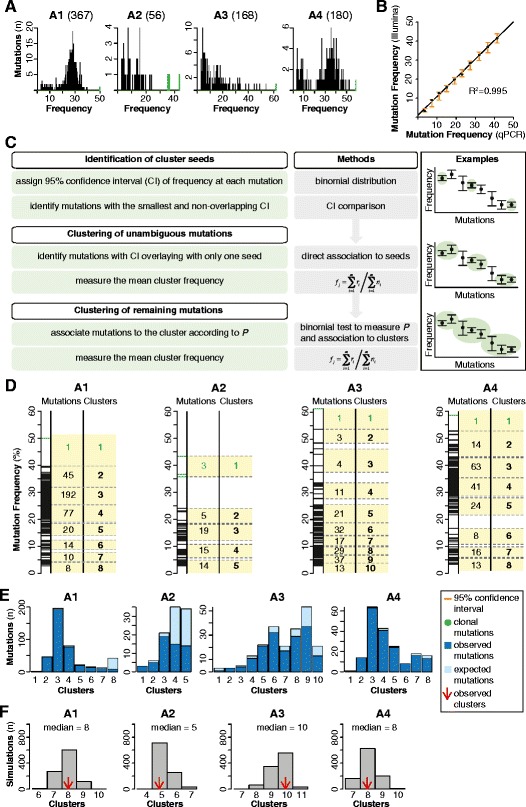
**Accuracy of Illumina frequency estimation and mutation clustering in the four tumors. (A)** Distribution of somatic mutations according to their frequency. Green bars represent clonal mutations. **(B)** Linear regression curve of the mutation frequency of 10 proportions of mutated allele measured with qPCR and Illumina sequencing. **(C)** Pipeline for mutation clustering. First, 95% confidence interval was assigned to each mutation. Second, mutations with non-overlapping confidence interval or, in case of overlap, with the smallest confidence interval, were identified as cluster seeds. Third, mutations unambiguously overlapping with only one seed were assigned to that seed. Finally, all mutations overlapping with more than one seed were assigned to a given cluster according to the highest binomial probability. **(D)** Clusters of mutations in the four samples. In all samples, clusters are highlighted in yellow and numbered progressively. For each cluster, the maximum, the minimum, and the number of mutations are shown. Green clusters contain clonal mutations. **(E)** Expected and observed somatic mutations for each cluster. The expected number of mutations per cluster was calculated as the number of observed mutations over the fraction of positions with coverage equal or higher than the minimum coverage for those positions. The number of observed mutations reflected that of expected mutations, except for low frequency mutations that were less than expected. These mutations were under-represented in our datasets likely because they are more difficult to identify and to distinguish from random errors. **(F)** Clustering performance. Shown are the distributions of the number of clusters obtained from 1,000 simulations. At each iteration, the frequency of 40% random mutations was varied within 95% confidence intervals, and mutations were re-clustered with our method. In all samples, the median of the distribution is equal to the observed number of clusters. Except for sample A3, the clustering of all other samples is robust even upon modification of higher percentage of mutations (Figure S4 in Additional file 2).

Although MSI CRCs are usually stable towards rearrangements and copy number alterations [21-23], we measured the copy number status of the X chromosomes to control for possible alterations that would affect the mutation frequency estimation. We selected eight regions broadly distributed along the entire length of the X chromosome (Figure S3 in Additional file 2) and assessed their copy numbers by TaqMan copy number variation assays. We observed no copy number variation in any of the regions in all analyzed tumors (Table S3 in Additional file 4), thus assessing the integrity of the X chromosome. These results also confirmed that all mutations that we detected were hemizygous because they lay on the X chromosome of male patients and no copy number variation was observed. Therefore, the frequency of each mutation directly reflected the fraction of mutated alleles in the tumor cell population and, hence, the fraction of mutated tumor cells. This ranged from all cells in the case of clonal mutations to less than 3% in the case of rare mutations present in a small portion of the cell population (Figure 4A and Table S1 in Additional file 1).

Because we inferred mutation frequency directly from Illumina reads and intended to use it for the reconstruction of the tumor proliferation trees, we evaluated Illumina accuracy in quantifying mutation frequency. To this aim, we quantified 10 dilutions (from approximately 4% to approximately 41%) of a homozygous germline mutation with the corresponding wild type genotype using qPCR. We then deep-sequenced each dilution with Illumina and measured the corresponding mutation frequency as the number of mutated reads over the total reads that covered that position. We found that qPCR and Illumina measures were highly comparable also for very low mutation frequencies (R^2^ = 0.995, Figure 4B and Table S4 in Additional file 5), thus confirming that Illumina deep sequencing is able to correctly measure mutation frequency. To account for the experimental error in the Illumina frequency estimations, we measured 95% confidence interval for each of the 10 dilutions using a binomial distribution with mean = *np*, where *n* was the observed depth of coverage obtained with Illumina sequencing and *p* was the frequency of the variant allele as measured by qPCR. We observed that the values of frequency measured with Illumina always fell within the 95% confidence intervals (Figure 4B), thus showing that the experimental uncertainty in Illumina frequency estimation can be modelled with a binomial distribution. Consequently, we accounted for the experimental error associated with the Illumina frequency of each mutations observed in the four tumors by measuring 95% confidence interval with a binomial distribution (Figure 4C). We used these confidence intervals to cluster mutations into discrete groups following a multi-step procedure (Figure 4C). First, we identified as cluster seeds mutations with either a confidence interval that did not overlap with that of any other mutation or, in case of overlap, with the smallest confidence interval. By definition, the seeds had the most accurate frequency estimations and defined the number of distinct clusters in each sample. Second, we assigned mutations to a given seed if the corresponding frequency confidence intervals unambiguously overlay only with that seed. Finally, we assigned all remaining mutations to the cluster with the highest probability measured using a binomial test (Figure 4C). At the end of this procedure, each cluster was composed of mutations whose frequencies were more similar to each other than to those of any other cluster (Figure 4D and Table S5 in Additional file 6). We verified that at least for high frequency mutations, the number of observed mutations per cluster was comparable to the expectation in all four samples (Figure 4E and Table S5 in Additional file 6). For low frequency mutations, instead, the observed mutations were fewer than expected, likely because these mutations require higher coverage to be detected with high confidence and are more difficult to distinguish from random errors. It should be noted, however, that low frequency mutations only populate clusters that correspond to the leaves of the trees and do not affect the inner branches (see below). The overall good correspondence between observed and expected number of mutations per cluster confirms that the obtained mutation profiles captured a representative portion of all somatic mutations that were progressively accumulated in the X chromosome during clonal expansion.

We further investigated clustering reliability and its dependence on the experimental errors of mutation frequency measurements. To this aim, we set up a simulation study where we randomly altered the frequency of a given fraction of the total mutations (from 10% to 100%) within its 95% confidence interval for 1,000 times. We then clustered mutations at each iteration and eventually obtained a distribution of clusters in the 1,000 simulations (Figure S4 in Additional file 2). Up to 40% of varied mutations, the majority of simulations (606 simulations for A1; 709 for A2; 557 for A3; 625 for A4) had the same number of clusters observed in the real data in all four samples (Figure 4F). The percentage of mutations that could be varied without detecting any difference between observed and expected clusters was even higher in samples A1, A2, and A4 (Figure S4 in Additional file 2). Finally, in order to exclude the possibility that the simulations were biased towards seeds, we confirmed that the seeds were not the only mutations with small confidence intervals (Figure S5 in Additional file 2). Altogether, these results indicate that the clustering method was robust even upon massive perturbation of mutation frequencies and succeeded in reliably grouping mutations with similar frequency.

### Tumor proliferation trees from mutation clusters

In order to infer the proliferation tree from the clusters of mutations, we relied on the model of tumor clonal evolution. The underlying assumptions of this model were that: (1) there is direct parent-descent relationship between cells of the tumor bulk; and (2) the only visible mutations in the final populations are those that survived the death of the whole cell lineage (Figure 1A and Extended Methods in Additional file 2). One direct consequence of this model is that each cluster identified from the four tumor profiles collected mutations present in a similar fraction of cells. In case of neutral evolution, these mutations also occurred at the same time of tumor expansion. In case of mutations acquired in cells under selection, no direct relationship could be inferred between frequency and insertion time. However, in either of the two scenarios, mutation frequency corresponded directly to the fraction of cells with that mutation because, for a mutation to be present in a given number of cells, a minimum number of divisions must have occurred. This number does not correspond to the exact number of cell divisions, rather it is based on the criterion of minimum evolution that minimizes the path of divisions needed to observe a given mutation frequency in the final population (Figure 1B). Another consequence of the parent-descent relationship between cells is that the mean frequency of each cluster can be directly exploited to identify the root, the external nodes (leaves) of the tree, and the path to connect them (Figure 1B). In particular, we identified the combination of nodes descending from the root and from all other clusters in each sample (N_*tot*_) by dividing the frequency of each cluster by the frequency of the lowest cluster (see Methods and Extended Methods in Additional file 2). It should be noted that this model does not imply that all mutations of one cluster occurred at the same time, but only that they are distributed among a defined number of nodes, which again recapitulate the minimum number of divisions that were needed to observe this frequency in the final population. The time for covering this path can be very different between each pair of parent-descend nodes depending on selection.

**Figure 5:**
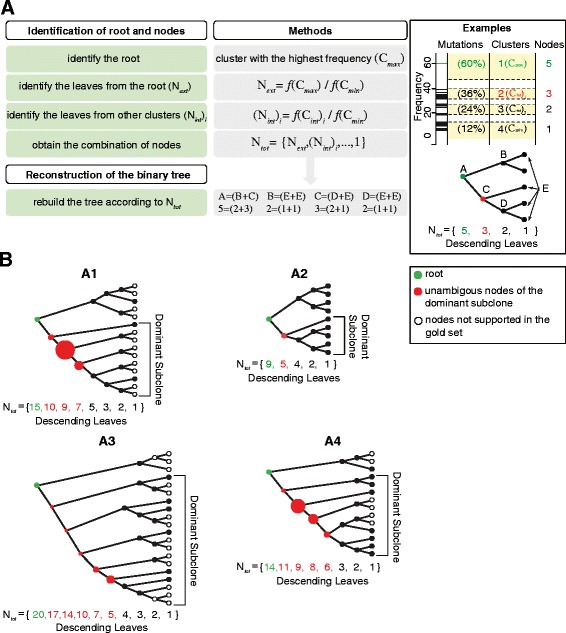
**Proliferation trees. (A)** Pipeline for tree reconstruction. Mean cluster frequencies were used to identify the root and to enumerate the external nodes (leaves) descending from each cluster. Once the combination of nodes (N_*tot*_) was identified for each tumor, the tree was rebuilt using a recursive algorithm. As explained in the text, the algorithm was based on the parent-descent relationship between nodes of a full binary tree, which resembles the parent-descent relationship between cells, and implies that each parent node led to two descending nodes. The algorithm started from the root of the tree and progressed down to the leaves by generating pairs of nodes according to the combination found in N_*tot*_. In the shown example, the first two nodes that directly descended from root A are node B, which led to two leaves, and node C, which leads to one leaf E and to node D. Node D, in turn, produces two leaves E. **(B)** Proliferation trees of the four samples. Each circle represents one node of the tree. In the dominant branch, mutations can be assigned to a given node (red) and the circle size is proportional to the number of mutations. Filled circles identify nodes supported by the gold sets (mutations with frequency ≥4% and in >6 different read positions). Of the four highly similar trees of sample A3 that were compatible with the obtained combination of nodes (Figure S6 in Additional file 2), only the one that makes no *a priori* assumption on the proliferation history is shown.

Starting from the combination of nodes N_*tot*_ that derived from the clusters of each sample, we developed a recursive algorithm that connected the nodes and reconstructed the corresponding proliferation tree compatible with N_*tot*_ (Figure 5A and Methods). For each tumor we rebuilt two trees, one using the clusters obtained from the entire set of mutations and the other using those obtained from the mutations of the gold set. Notably, the two trees were always identical at the main branches and minor differences, if any, were detected only at the leaves of the trees (Figure 5B and Figure S6 in Additional file 2). This confirms that even if we detected a number of low frequency mutations lower than expected (Figure 4E), this did not affect the main branches of the trees. In addition, it also shows that also the frequency of very rare mutations is reliably estimated, although we had no orthogonal method to validate it.

### Robustness of proliferation tree reconstruction

We have several indications that our method led to a robust tree reconstruction. First, in all four samples we could always identify at least one binary tree that was compatible with N_*tot*_, and this is not expected to occur by chance (*P* =1.1 × 10^-4^, see Methods). Second, we identified only one tree in samples A1, A2, and A4 and four almost identical trees in sample A3, despite there was a high number of possible trees that were compatible with the observed number of external nodes (ranging from 286 trees for sample A2 to more than 12,000,000 trees for sample A3 [2,32], see Methods). Third, we performed a series of simulations in which we randomly removed a given fraction of mutations (from 1% to 20% of the total) for 1,000 times, clustered the remaining mutations and rebuilt the corresponding trees. At the end of the simulations, we counted how many different combinations of nodes were obtained. For both the gold and the whole sets of mutations, the combination of nodes observed in real data was also the most frequent in the simulations (Figure S7 in Additional file 2). In addition, the second most frequent combination differed from the observed one only for one node. These results show that our tree reconstruction was stable also upon removal of a substantial fraction of mutations and that the signal contained in the tumor mutation profile of each adenoma was robust enough to allow the clear and unambiguous identification of the corresponding proliferation tree.

### Properties of the four proliferation trees

One recurrent feature shared in all four trees was their asymmetric growth meaning that one of the two branches of the tree contributed to the final population more than the other (red dots in Figure 5B). Notably, this feature was present also in sample A2, despite this tree was poorly informative due to the low number of mutations. These results suggest that the majority of the tumor cell population is composed of one dominant subclone that prevailed over the others from the early phases of tumor progression and that cells of the tumor bulk have different proliferative potential. Therefore, the mutation profile of each tumor harbors the traces of the selection that occurred during clonal expansion. Next, we analyzed the distribution of mutations along the branches of each tree and noticed that a variable number of mutations was introduced in the different nodes of the four samples (Figures 4D and 5B). The uneven distribution of mutations among the nodes can be biologically explained with the occurrence of cell death and/or asymmetrical cell divisions at that point of tumor growth. If after cell division only one daughter cell survives and maintains the capability of transferring the genetic information to the progeny, the total number of cells for that cycle will not increase and mutations accumulated at that stage of tumor development will have similar frequency in the final population. Therefore, the number of mutations in the nodes may provide useful information on the tumor growth history. In samples A1 and A4, more than 80% of all somatic mutations could be unambiguously assigned to the early nodes of the dominant branch (Figure 5B). This suggests that the initial growth of these tumors was not exponential; rather the dominant subclone required a certain amount of time before eventually prevailing. Mutations in sample A3 were instead more evenly distributed along the dominant branch and the corresponding tree showed a dense branching already from the early phases (Figure 5B). These features are compatible with a rapid establishment of the dominant subclone and with a sudden initiation of tumor proliferation. Although no inference is possible for sample A2 because there is only one unambiguous node of the dominant branch, the comparative analysis of the other three tumors indicates that the growth potential of the dominant subclone is highly variable among samples.

## Discussion

Our study aimed at rebuilding the proliferation history of four adenomas from the mutation profile of the X chromosome. The trees that we obtained show that each tumor is a dynamic system that grows following a specific path. One recurrent feature of the proliferation trees of all four tumors is the asymmetric growth that can be explained with the progressive establishment of a dominant subclone that composes most of the tumor bulk (red dots of Figure 5B), while the rest of the mass is formed of minor subclones (black dots). It should be noted that, since we only sequenced the X chromosome of four patients, driver mutations responsible for the selective advantages of the dominant subclone are unlikely to be among the mutations that we identified and used for the tree reconstruction. Nonetheless, we were able to detect different selection pressures acting on sister branches of the tree and the appearance of the dominant subclone in one of them. Our method does not require the presence of driver mutations among the ones used for tree reconstruction: when one or more driver mutations occur somewhere in the cancer genome and give selective advantages, this is reflected in the number and frequency of the detected mutations in the final population and, eventually, in the obtained tree.

The genetic heterogeneity between dominant and minor subclones of the four tumors suggests functional heterogeneity among the cells that constitute the bulk of solid tumors, similarly to what has been shown for breast and CRC [11,33], leukemia [34,35], and multiple myeloma [13]. Heterogeneity of the colorectal tumor bulk has been so far ascertained through the isolation of colon cells that express stem-like properties [36,37], through the capacity of primary CRC to differentiate into multiple lineages [38], and through the identification of transcriptional heterogeneity [39]. We now add the formal evidence that cells of MMR-deficient tumors are genetically heterogeneous and do not contribute equally to the initial phases of tumor growth.

Despite the common hierarchical organization, the four proliferation trees show a high variability in their evolutionary history, as shown by the uneven distribution of mutations among the nodes in the four trees (Figure 5B). There are several reasons that likely concur to this variability. The proliferation history could depend on the tumor-specific landscape of driver mutations that confer a variable degree of selective advantage. Large-scale resequencing screenings of cancer genomes have so far identified 2,000 mutated genes that potentially play an active role during cancer development [31,40,41]. Except for key genetic players that are recurrently mutated (*TP53*, *PIK3CA*, *PTEN*, *KRAS*, and a few others), the large majority of driver genes are tumor-specific. Thus, since the mutation landscape in cancer is specific of each tumor type and sample [42,43], also the proliferation history will be sample specific. Our results suggesting a sample-specific proliferation history well agree with a scenario where each cancer type, if not each lesion, has its own repertoire of mutations that directly influence the way that particular tumor developed. This scenario would suggest that the multistep progression may be needed not only for the adenoma to carcinoma transition [15] but also for the establishment of the dominant subclone in the early phases of tumor progression. In addition to the genetic contribution, the variable proliferation history could also be due to the macro- and micro-environment that surrounds and sustains tumor growth. For example, external factors such as inflammation are known to support the acquisition of genetic mutations during cancer progression [44] and they could play a role in determining the growth dynamics. Another intriguing reason for the variable proliferation history may reside in the distinct molecular mechanisms that promote tumorigenesis in different types of MMR-deficient tumors. In our analysis, sample A3 is the only sporadic adenoma with hypermethylation of *MLH1* and a non-synonymous mutation in *BRAF* [45]. Samples A1, A2, and A4 are instead hereditary non-polyposis colorectal lesions with heterozygous germline mutations in the MMR genes [46]. Sporadic and inherited MSI CRCs have heterogeneous clinical and molecular features, including age of diagnosis and different polyp morphologies [47]. Accordingly, it is not surprising that these two tumor types also show different proliferation histories. Moreover, sample A3 derives from an older patient and age at diagnosis might also influence the evolution of the tumor clone. Even though referred only to one case of sporadic MMR-deficient tumor, our data suggest that the outgrowth of the malignant component from a mismatch deficient polyp with hMLH1 methylation is a fast event. Such fast growth in the presence of *BRAF* mutations can be hypothesized to be relevant in the progression of right-sided colon tumors, which are difficult to timely diagnose and effectively prevent [48]. This is compatible with the adverse outcome of sporadic MMR-deficient CRCs with a *BRAF* V600E mutation [49,50]. To support this hypothesis, the growth dynamics of more samples of the two cancer types is needed. If confirmed, this difference not only will open new scenarios to our comprehension of the molecular basis of these two CRC types, but could also directly impinge on patients, and in particular on the selection of the best therapeutic approach.

The experimental conditions that we used, in particular the size of the genomic region and the depth of coverage, allowed a robust tree reconstruction for lesions with high degree of genomic instability, both for frozen and FFPE samples (A1, A3, and A4). Sample A2, which was almost stable and had few somatic mutations, showed a tree with few nodes that was poorly informative on the proliferation history. To apply this approach also to mutationally stable lesions, a larger region needs to be sequenced at deeper coverage in order to collect a number of mutations large enough to rebuild an informative tree.

## Conclusions

Our analysis of four mismatch repair deficient colorectal adenomas confirmed the evidence that clonal evolution is a highly heterogeneous process where different tumor lesions grow following distinct evolutionary histories. These differences are likely depending on a combination of genetic and environmental factors that changed in each patient or lesion. The uneven distribution of mutations among the nodes of the four proliferation trees suggests the co-existence of different subpopulations of cells, which may contribute to the overall evolution of the disease. Our findings shed light on the main patterns of evolution that happen during the tumoral subclone establishment in individual lesions. With the advent of personalized medicine, it is not far the time when the genetic signature will be easily derived for each single lesion of a patient and used to predict tumor progression, thus providing a useful support for the cure selection.

## Methods

### Sample description

Samples used in the study (A1, A2, A3, A4) derived from four male individuals cured at Centro di Riferimento Oncologico, Aviano, Italy and Istituto Clinico Humanitas, Rozzano, Milan, Italy. All patients signed a written consent for research and dissemination of results in compliance with the Helsinki Declaration (CRO-15-97,29/04/1997 and ICH-25-09, 07/05/2009). A1 was a frozen sample, while A2, A3, A4 were formalin-fixed paraffin-embedded (FFPE) samples. The blood (A1 and A4) and the normal FFPE tissue (A2 and A3) were used as matching normal reference. *KRAS* exons 2 and 3 (containing codons 12, 13, and 61 that are frequently mutated in CRC) were amplified and Sanger sequenced in DNA from each tumor and corresponding normal counterpart. PCRs were performed using Taq polymerase (Genespin) or GoTaq Master Mix (Promega), purified with ExoSap-it (USB Products, Affymetrix), and sequenced using the ABI PRISM 310 Genetic Analyzer (Applied Biosystems). In samples A2, A3, and A4, BRAF V600E mutation was screened using TaqMan SNP Genotyping Assay (Applied Biosystem). For sample A1, *BRAF* exon 15 was amplified and Sanger sequenced in DNA from tumors and corresponding normal counterparts. MSH2 protein expression was checked via immunohistochemistry. Three micromillimeter-thick sections were cut, deparaffinased, rehydrated, immersed in an antigen retrieval solution (Diva Decloaker, Biocare Medical) and incubated in the Decloaking Chamber pressure system for 3 min at 125°C and then 5 min at 90°C. Subsequently, the endogenous peroxidase activity was quenched using the Peroxidase-1 (Biocare Medical) for 10 min and the non-specificities blocked by means of the Background Sniper (Biocare Medical) for 20 min at room temperature. The slides were treated for 1 h at room temperature with primary antibodies raised against MSH2 (clone FE11, 1:200, Calbiochem) and subsequently incubated with a polymer (MACH 4 Universal HRP-Polymer, Biocare Medical) for 30 min. 3,3-diaminobenzidine tetrahydrochloride (Dako) was used as a chromogen to yield brown reaction products. The nuclei were lightly counterstained with hematoxylin solution. The lack of expression of MSH2 was assessed in all tissues under an optical microscope by two histologists, independently. Sample features are described in Table 1.

### Sample preparation, target enrichment, and Illumina sequencing

Genomic DNA was extracted using the DNAeasy tissue kit (Qiagen) according to the manufacturer’s protocol for sample A1 and corresponding blood. For samples A2, A3, and A4 and FFPE normal counterparts, paraffin was removed and DNA was then extracted using the proteinase K digestion and phenol-chloroform purification. In each sample, around 2 Mbp exonic regions corresponding to 921 genes and around 15 Mbp intergenic regions were targeted using the SureSelect Human X Chromosome Panel kit (Agilent) and two SureSelect custom kits, respectively. Sequence repeats, segmental duplications, PAR regions, and gaps in the genome assembly were excluded from the design of the custom kits. In addition, only continuous regions longer than 200 bp and with GC content ranging from 30% to 65% were selected to optimize the capture efficiency [24,25]. Target capture and sequencing were done following the manufacturer’s protocol with slight modifications. Briefly, a variable amount of genomic DNA ranging from 3 to 13 μg was sheared using an ultrasonic disruptor (Bioruptor, Diagenode). After library preparation with Illumina Paired-End DNA Sample Prep Kit, 150 to 300 bp fragments were selected and purified by gel extraction. Fragments were further amplified with 10 to 14 cycles of PCR and 500 ng were hybridized with each bait library. DNA capture was followed by paired-read cluster generation on the Cluster Station (Illumina). The obtained libraries were sequenced on the Genome Analyzer IIx with the 76 paired end protocol, using six to nine lanes for each tumor sample and two lanes for the matching normal sample of A1. The libraries obtained from the normal counterparts of A2, A3, and A4 were sequenced using two lanes of Illumina HiSeq2000 per sample, with the 101 bp paired-end protocol.

### Pipeline for variant calling

Paired-end reads were mapped to the human genome (NCBI36/hg18) using Novoalign [51] allowing a maximum of three mismatches per read. All reads uniquely mapping within 75 bp of the targeted regions were considered on target and retained. A novel approach was developed to call variants spanning a broad range of frequencies (Figure 2B). As a first step, variant sites were considered for further analysis if they were: (1) supported by at least 2 high quality mismatches both in forward and reverse reads (phred quality score ≥30); and (2) located in regions of the read where the cumulative number of mismatches increased linearly. The first filter discarded amplification errors that tend to recur always in the same position of identical reads as well as variant base calls supported by poor quality scores. The second filter allowed the removal of regions of the reads that accumulate most sequencing and alignment errors (Figure 2C and D). Further statistical tests were applied to remove multiple sources of error. First, the quality score and the coverage of all aligned bases at each variant site were used to calculate the probability that *r* variant base calls out of *n* total base calls (coverage) were due to sequencing errors. Each base call *i* was assumed as a Bernoulli random variable with success probability *p*_*i*_ = 10^-0.1x*Qi*^, where *Q*_*i*_ is the quality score of base call *i*. The mean probability for the sum of *n* total base calls was measured as:

$$ \mu \kern0.5em =\kern0.5em {\varSigma}_i\kern0.5em {p}_i $$

The upper bound on the *P* of observing *r* base calls was inferred using the Chernoff bound [52] for any δ >0, similarly to [53]:

$$ P\left[X>\kern0.5em \left(1\kern0.5em +\kern0.5em \delta \right)\kern0.5em \mu \right]\kern0.5em <{\left[\frac{e^{\delta }}{{\left(1\kern0.5em +\kern0.5em \delta \right)}^{1\kern0.5em +\kern0.5em \delta }}\right]}^{\mu } $$

where *r* = (1+ δ)μ.

Second, the propensity to accumulate errors was measured in the region around each variant site. To this aim, the occurrence of the reference base calls at five and 10 flanking sites were averaged to compute the expected mean of a binomial distribution. The *P* to test that the occurrence of reference base calls at the variant site is higher than at the flanking sites was computed using a binomial one-tailed test [4].

Each test was performed on forward and reverse reads independently, and the resulting four *P*s were adjusted for the number of variant sites in each sample (9,717 variant sites for A1; 7,681 for A2; 10,632 for A3; and 10,594 for A4) using Bonferroni correction. Variant sites were retained if they showed adjusted *P* <0.01 in each test and their frequency was calculated as the number of variant base calls divided by the coverage at each site. SNPs were identified after comparison with the normal counterpart and with several databases (dbSNP130 and dbSNP131, 1000 genomes project and six personal genomes). The lower bound of mutation frequency was set to 2.7%, which is the minimum frequency that was found in all four samples. The final set of somatic mutations with a depth of coverage >50× underwent further manual inspection and the possible effects on coding sites were predicted using SIFT [54]. In all samples, gold sets of mutations with frequency >4% (lower limit of the TaqMan assay) and supported by more than six reads starting at different genomic positions were identified (Table S1 in Additional file 1).

Somatic indels were identified using VarScan2 [55] and after comparison of each tumor with the normal counterpart. All somatic indels were absent in the normal sample, covered by at least 10 reads, and with frequency ≥20%. The obtained pool underwent manual inspection for further check.

### Variant validation with orthogonal methods

For mutations with frequency higher than 9%, genomic DNA from tumor samples was amplified by PCR using the Taq DNA Polymerase (New England BioLabs) and sequenced with Sanger in both directions on a 3730xl DNA Analyzer (Applied Biosystems) using the dRhodamine chemistry. Mutations with frequency ranging from 4% to 9% were validated using TaqMan SNP Genotyping Assays, according to the manufacturer’s protocol, using the ABI Prism 7900HT Sequence Detection System (Applied Biosystems). In sample A1, an additional set of mutations with frequency ranging from 10% to 31% was also validated with both TaqMan and Sanger sequencing to prove the reliability of the latter to detect mutations in this range of frequency.

### Analysis of copy-number variation

Copy number status was assessed by quantitative RT-PCR using the TaqMan Copy Number Assay on a 7900HT Fast Real-Time PCR System (Applied Biosystems) and the Sequence Detection Systems Software 2.2.2. Eight pre-designed TaqMan probes were selected within the targeted regions, in proximity of cluster seeds, and broadly distributed on the entire length of X chromosome (Figure S3 in Additional file 2 and Table S3 in Additional file 4). Copy Number Reference Assay TERT (Applied Biosystems, part number 4403316) was used as a reference. Samples A1, A3, and A4 were plated in quadruplicates using approximately 20 ng of DNA for each reaction. Sample A2 could not be tested because no DNA was available for this sample. Copy-number calling was done with CopyCaller v2.0 (Applied Biosystems), using the matched normal counterpart as calibrator. In case of low confidence value for the copy number assignment, data were reanalyzed using the mean delta cycle threshold of all samples as a calibrator (Table S3 in Additional file 4). High degradation of sample A3 and matched normal N3 resulted in higher Ct values, as known for FFPE samples [56]. Sample A3 was therefore analyzed separately and no confidence could be assigned to the detected copy number owing to the low number of samples.

### Controlled dilutions for assessing Illumina accuracy

Controlled dilutions were performed using a murine 105 bp long region located on chromosome 5 and carrying a single nucleotide polymorphism C/T (SNP rs32609672, dbSNP build 128). The region was amplified from the genomic DNA of two mice with CC and TT homozygous genotypes (FVB/NJ and C57BL/6 J strains), using a nested PCR approach. First, a 393 bp fragment was amplified from each genomic DNA and sequenced with Sanger for genotype confirmation. Subsequently, a nested PCR was performed to generate the 105 bp long amplicon, centered on the base of interest. The two amplicons (CC and TT) were purified using the MinElute PCR Purification Kit (Qiagen) and used for library preparation with Illumina Paired-End DNA Sample Prep Kit. The two libraries were pooled in 10 different molar ratios with C:T proportion ranging from 0.04 to 0.41 (Table S4 in Additional file 5). The concentration of the two alleles in each pool was quantified by qPCR and was used to compute the expected values of mutation frequency in the dilution curve. In order to reach a depth of coverage comparable to the tumor samples, each murine pool was sequenced on a different Illumina GAIIx lane together with a human sample in an approximately 1:1,000 molar ratio. The obtained reads were aligned to the mouse chromosome 5 (NCBI37/mm9) using Novoalign [51] allowing a maximum of three mismatches per read.

### Direct mutation clustering

Ninety-five percent confidence interval of frequency was measured for each mutation given the coverage for that position, using a binomial distribution. In this way, possible uncertainties in frequency estimation due to the position coverage were taken into account (Figure 4B). In each sample, cluster seeds were defined as mutations with non-overlapping confidence intervals or, in case of overlap, with the smallest confidence interval. Seeds had the most accurate frequency estimation and defined the number of distinct clusters for each sample. Starting from the seeds, mutations were clustered in a two-step procedure. First, all mutations with a confidence interval overlaying with only one seed were unambiguously associated to that seed. The frequency of unambiguous clusters was calculated as:

$$ {f}_i\kern0.5em =\kern0.5em {\displaystyle \sum_{i\kern0.5em =\kern0.5em 1}^m{r}_i}\kern0.5em /{\displaystyle \sum_{i\kern0.5em =\kern0.5em 1}^m{n}_i} $$

where *m* is the number of unambiguous mutations of cluster *j*, and *r*_*i*_ and *n*_*i*_ are the number of variant base calls and the coverage for mutation *i*, respectively. Second, all remaining mutations were assigned to the cluster with the highest *P*, measured with the binomial test that compared the frequency of each mutation with the frequency of each unambiguous cluster. The frequency of the final clusters was calculated as before, using the total pool of mutations. This frequency was directly used for identifying the node combinations of the trees. Clustering was done independently for the mutations of the gold set and for all mutations (Table S5 in Additional file 6).

### Reconstruction of proliferation trees

To rebuild the proliferation trees directly from the mutation clusters, we relied on the model of cancer clonal expansion that represents tumor evolution as a fully binary tree (Figure 1B). This model is based on two assumptions:

- Assumption 1: there is direct parent-descent relationship between tumor cells. This assumption implies that: (1) daughter cells inherit all mutations from the parent, acquire new ones, and pass old and new mutations to the progeny; and (2) subclones are not independent because all of them derive from the same cell of origin.
- Assumption 2: the only mutations that are detectable in the final population are the ones that survive the death of the whole cell lineage. This implies that: (1) only the proliferation history of subclones that survived extinctions can be reconstructed; and (2) no assumption can be made on subclones that died out, because they did not leave traces (that is, mutations).

One direct consequence of the parent-descent relationship between cells is that the total number of external nodes (leaves) that descend from the root (N_*ext*_) can be counted as:

$$ {N}_{ext}=\kern0.5em f\kern0.5em \left({C}_{max}\right)/f\kern0.5em \left({C}_{min}\right) $$

where *f*(C_max_) and *f*(C_min_) are the frequencies of the highest and lowest clusters, respectively. In our samples, N_*ext*_ was 15, 9, 20, and 14 for A1, A2, A3, and A4, respectively (Table S5 in Additional file 6). The number of possible rooted binary trees increases with N_*ext*_ and can be identified using the Wedderburn-Etherington numbers [57,58]. Given the number of external nodes, the four analyzed samples were compatible with 87,811, 286, 12,826,228, and 32,973 different binary trees, respectively [2,32].

Similarly, the number of leaves (N_*int*_)_i_ that descend from each other cluster (C_*int*_)_i_ is:

$$ {\left({N}_{int}\right)}_i\kern0.5em =\kern0.5em f{\left({C}_{int}\right)}_i/f\left({C}_{\min}\right) $$

where *f* (C_*int*_)_i_ represents the frequency of cluster *i*.

At the end of this procedure, the total combination of leaves that descend from each cluster (N_*tot*_) is:

$$ {N}_{tot} = \left\{{N}_{ext},\ {\left({N}_{int}\right)}_i, \dots,\ 1\right\} $$

where 1 corresponds to *f*(C_min_) / *f*(C_min_).

The tree was directly inferred from the combination N_*tot*_ using a recursive algorithm based on the parent-descent relationship between nodes of a full binary tree, which resembles the parent-descent relationship between cells. This relationship implied that each parent node led to two descendant nodes. The algorithm started from the root of the tree and progressed down to the leaves by generating pairs of nodes according to the combination found in N_*tot*_, as shown in Figure 5A. For each adenoma, we always obtained at least one binary tree and all possible trees for the gold set and for all mutations are reported in Figure S6 in Additional file 2.

Since not all combinations of descending nodes may be connected to form a binary tree, we measured the probability to obtain a binary tree given the observed combination of nodes in all four samples. All possible combinations of internal nodes were enumerated in each sample as S_*n,c*_ = *c*!/*c*!(*n-c*!), where *n* is the number of possible values associated to the internal nodes and *c* is the number of corresponding clusters. These values are within 2 and N_*ext*_-1. Each random combination of nodes N_*r*_ was used for verifying the existence of a tree with T = {N_*ext*_, N _*r*_, 1} and the number of successful combinations was counted. *P* was calculated as the fraction of successful combinations out of the total trials in the four samples.

### Data availability

Sequence data for all samples are available from the European Genome-phenome Archive [EGA:EGAS00001000883].

## Additional files

Additional file 1:
**Mutation profile of the four samples.** This file contains the supplementary Table S1 including the mutational profile of analyzed each tumor. Additional file 2:
**Supplementary information and Supplementary figures.** This file contains the information about the model assumption, tree reconstruction and interpretation. Supplementary figures S1 to S7 are also included. Additional file 3:
**Indels present in the four samples.** This file contains the supplementary Table S2 including detailed information for all indels found in each tumor. Additional file 4:
**TaqMan copy number assays of each tumor.** This file contains the supplementary Table S3 including the results of TaqMan copy number assays that assess the integrity of the X chromosome in the targeted regions shown in Additional file 2: Figure S3. Additional file 5:
**Controlled dilution experiment.** This file contains the supplementary Table S4 including the results of the controlled dilution experiment. Additional file 6:
**Clustering for the mutations of each sample.** This file contains the supplementary Table S5 summarizing all clustering steps described in Figure 4 and that are required to build the proliferation trees described in Figure 5.

## Abbreviations

FFPE: Formalin-fixed paraffin embedded
Indel: Small insertion or deletion
MMR: Mismatch repair
NGS: Next generation sequencing
SNP: Single nucleotide polymorphism
