# Supplementary material for: Deep sequencing of the X chromosome reveals the proliferation history of colorectal adenomas
Source: Genome Biol. 2014 Aug 30;15(8):437. doi: 10.1186/s13059-014-0437-8 (PMC4181412; doi:10.1186/s13059-014-0437-8)
Supplement: Additional file 2: — Supplementary information and Supplementary figures. This file contains the information about the model assumption, tree reconstruction and interpretation. Supplementary figures S1 to S7 are also included. [file 13059_2014_437_MOESM2_ESM.pdf]

Deep sequencing of the X chromosome reveals the history of colorectal adenomas

Anna De Grassi[1,*,#], Fabio Iannelli[1,*], Matteo Cereda[1,2], Sara Volorio[3], Valentina Melocchi[1], Alessandra Viel[4], Gianluca Basso[5], Luigi Laghi[5], Michele Caselle[6], Francesca D. Ciccarelli[1,2,§]

[1]Department of Experimental Oncology, European Institute of Oncology (IEO), 20139 Milan, Italy

[2]Division of Cancer Studies, King's College London, London SE1 1UL, UK

[3]Cogentech-IFOM Istituto FIRC di Oncologia Molecolare 20139 Milan, Italy

[4]CRO Aviano National Cancer Institute, 33081 Aviano (PN), Italy

[5]Laboratory of Molecular Gastroenterology, Department of Gastroenterology, Humanitas Clinical and Research Center, Via Manzoni 56, 20089 Rozzano (MI), Italy

[6]Department of Theoretical Physics and INFN University of Turin, 10125 Turin, Italy

# present address: Department Biosciences, Biotechnology and Biopharmaceutics, University of Bari, 70125 Bari, Italy

*These authors contributed equally to this work

[§]Corresponding author

Email address: francesca.ciccarelli@kcl.ac.uk

TABLE OF CONTENT:

Extended Methods

Supplemental Figures

EXTENDED METHODS

Model assumptions and tree reconstruction

Our method relies on the model of tumor clonal expansion and is based on two main assumptions:

Assumption 1: there is direct parent-descent relationship between tumor cells. This assumption implies that (1) daughter cells inherit all mutations from the parent, acquire new ones, and pass old and new mutations to the progeny, and (2) subclones are not independent because all of them derive from the same cell of origin.

Assumption 2: the only mutations visible in the final populations are the ones that survive the death of the whole cell lineage. This implies that (1) only the proliferation history of subclones that survived extinctions can be reconstructed, and (2) no reconstruction can be done for subclones that completely died out, because they did not leave traces in the form of mutations.

According to this model, the frequency of each mutation represents the fraction of cells that bear that mutation: the higher the fraction of cells the higher the mutation frequency. The collection of mutations of a tumor population can be therefore used to rebuild the proliferation tree of the tumor in the form of a fully binary tree. It should be noted that this method does not require the presence of driver mutations in the pool of mutations used for the tree reconstruction. If one or more driver mutations occur somewhere in the cancer genome, the resulting selective advantage will be reflected in the number and frequency of mutations that are accumulated also in the X chromosome.

In order to rebuild the tree, three main steps are required:

1- Collect clonal and subclonal mutations that were progressively acquired during clonal expansion and that constitute the mutation profile of the tumor (see Methods and Figure 2B);

2- Cluster mutations into discrete groups according to their frequencies (see Methods and Figure 4C);

3- Rebuild the tree from the mutation clusters, starting from the combinations of nodes and using a recursive algorithm (see Methods and Figure 5A).

The relationships between mutations, clusters, and nodes in each tree are summarized in the figure below, which shows a toy sample whose mutation profile is composed of 41 mutations with different frequencies:

TOY TREE DERIVED FROM A MUTATION PROFILE OF 41 MUTATIONS

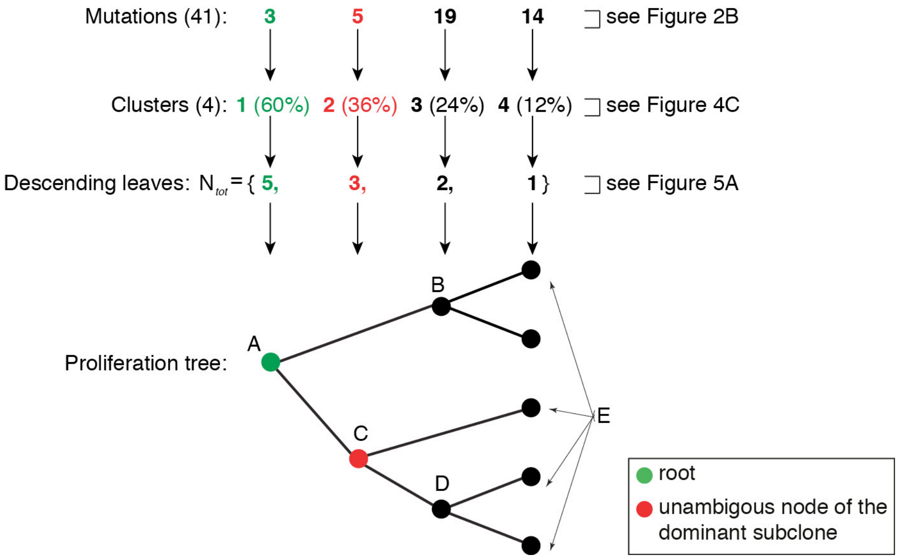

First, mutations are grouped according to their frequencies into four clusters, containing 3, 5, 19, and 14 mutations, respectively. The frequency of each cluster is measured as the mean frequency of all mutations present in that cluster (60%, 36%, 24%, and 12%). Second, the leaves descending from the four clusters are derived dividing the frequency of each cluster by the frequency of the lowest cluster and they correspond to 5, 3, 2, and 1. Finally, all possible full binary trees that are compatible with the obtained combination of leaves are

rebuilt. In the toy model there is only one compatible tree, as shown in the picture. Since we assume a model of tumor clonal expansion with a direct parent-descent relationship between tumor cells (Figure 1B), the algorithm that we implemented only searches for full binary trees that are compatible with the combination of descending leaves reported in N_{tot}.

Interpretation of the tree

(1) Each node of the tree indicates the points during clonal expansion where a given mutation *was introduced* and, from that point on, in which node(s) *it was inherited*. For example, nodes B and C of the toy model both derived from node A and both inherited all 3mutations already present in that node. All nodes that descend from B and C (nodes D and leaves E) will inherit the three mutations in addition to the mutations acquired in node C and B, respectively. In case one cluster corresponds to a single node, mutations can be unambiguously assigned to that node (red node C in the example). When more than one node is associated with one cluster, mutations introduced at that level cannot be unambiguously associated with a specific node (*i.e.* mutations introduced in nodes B and D cannot be distinguished).

(2) The tree is able to capture the competition between cells and the different selection acting on sister nodes of the tree. Let us consider again the toy tree reported in the figure: node B is the sister node of node C, which is the dominant subclone. The sisterhood relationship means that they both derived from the same parent node A (the root in this case). Despite the common origin, the mutations introduced in node B have lower frequency in the population than those introduced in node C. The biological explanation for this difference is that node B is under a selective pressure lower than the dominant subclone and therefore it gave rise to a lower fraction of cells in the final population.

(3) Our model does not account for the case where the mutations *introduced* in a child node have frequency higher than those *inherited* from the parent node. This case violates the direct parent-child relationship where inherited mutations have frequency always higher than those acquired because the former will always have at least one cell division more than the latter. Let us refer once again to toy tree: the mutations introduced in node C will always have lower frequency than those inherited from the root, although node C is under selection.

SUPPLEMENTAL FIGURES

Figure S1. Examples of validated mutations.

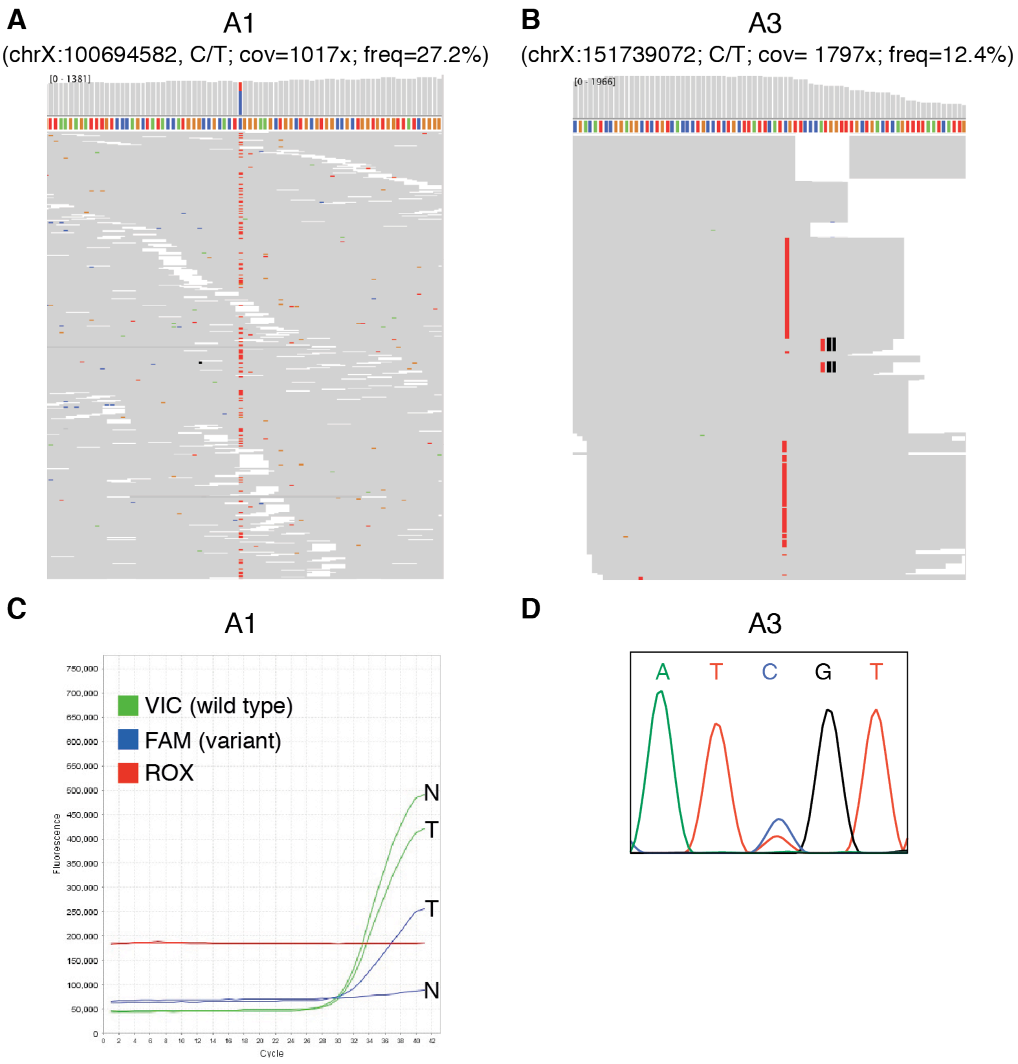

Screenshots of aligned reads bearing the mutated C and the wild type T bases are shown for two mutated sites in A1 (A) and A3 (B), respectively. In the first case, the mutated base is present in 73 different read positions, while in the second it is present only in six positions. Both mutations are true, as confirmed by the TaqMan assay (C) and Sanger electropherogram (D). The occurrence of mutations present in few positions of the reads is particularly frequent in samples A2 (30% of mutations) and A3 (48% of mutations), and is likely due to DNA fragmentation. To account for the possibility that these mutations, although true, have inaccurate frequency estimation due to PCR amplification, we eliminated them from the gold

set. It should be noted, however, that the trees rebuilt using the gold sets are always compatible with those rebuilt using the whole set of mutations (Figure 5B and Figure S5).

Figure S2. Probes for copy number assay.

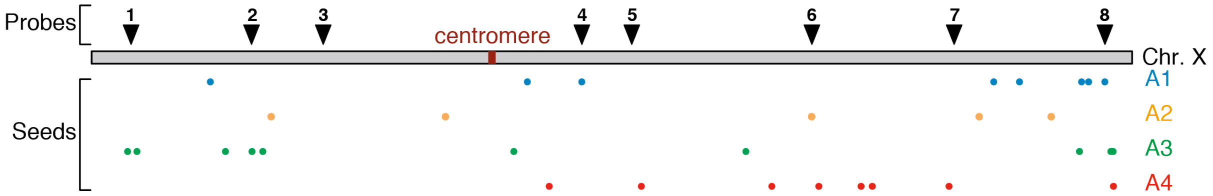

Eight probes were selected in order to be within the targeted regions of the X chromosome and broadly distributed along its entire length. These probes also fell in proximity of the cluster seeds of the four tumors to check for copy number status of regions essential for mutation clustering and tree reconstruction.

Figure S3. Assessment of clustering performance.

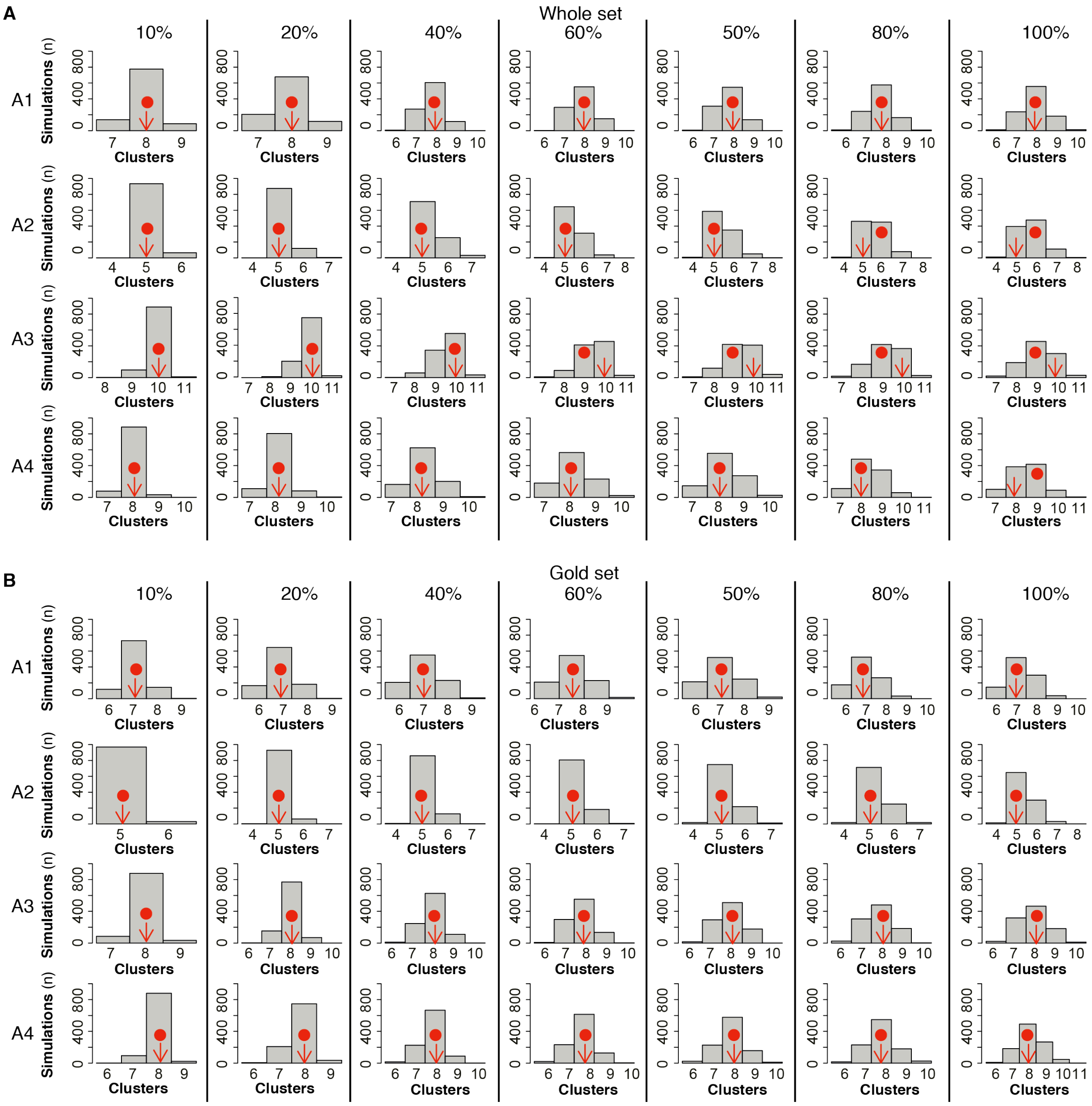

In each sample, the frequency of a variable fraction of mutations (from 10% to 100%) was varied within 95% confidence intervals in the whole (A) and in the gold (B) sets of mutations. Mutations were then re-clustered with our method. In all samples, the median of the distribution of the obtained number of clusters (red dot) was equal to the observed number of clusters (red arrow) up to 40% of varied random mutations. For samples A1, A2, and A4 the clustering of all other samples resulted to be robust even upon modification of 100%, 50%, and 80% of mutations, respectively.

Figure S4. Confidence interval comparison between cluster seeds and the rest of variants.

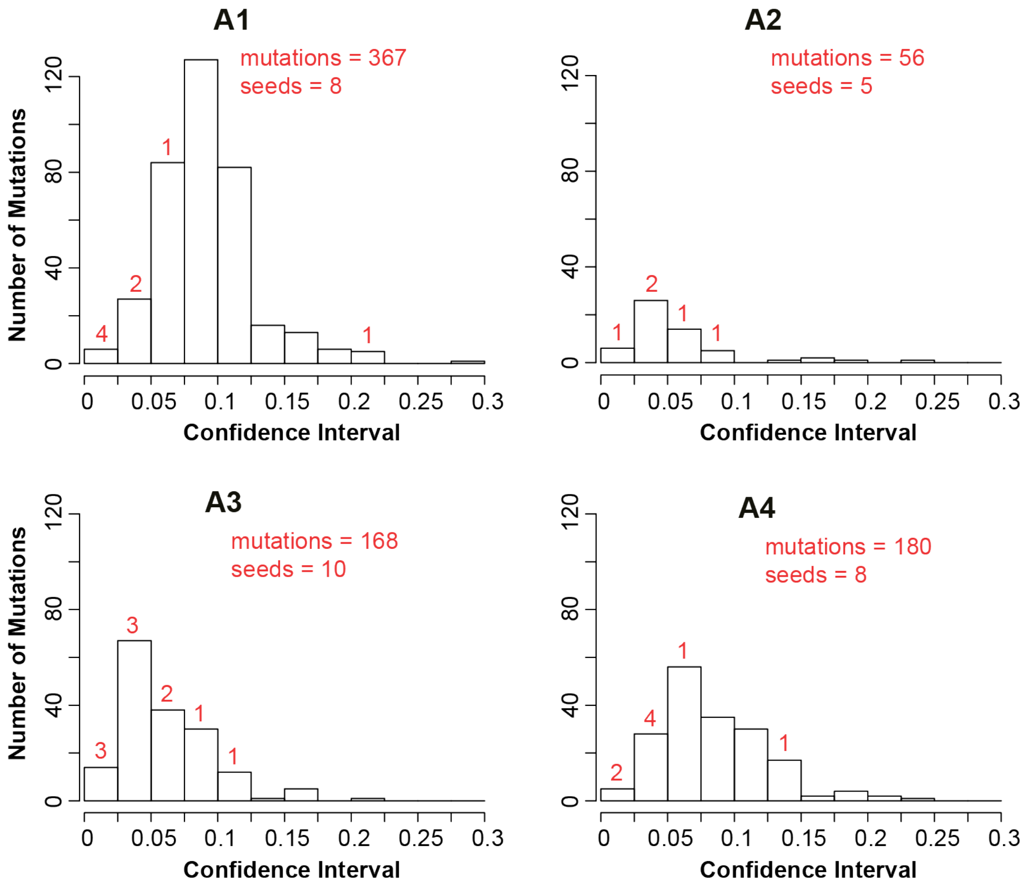

For each sample, mutations were grouped into bins of confidence intervals (*i.e.* from 0 to 0.025; from 0.025 to 0.05; etc). The number of seeds that fall into each bin is reported in red. As expected, seeds have small confidence intervals but there are also several other mutations with confidence intervals comparable to those of the seeds, thus excluding that our simulations were biased towards the seeds.

Figure S5. Tree Topologies of Gold and Whole Sets of Mutations.

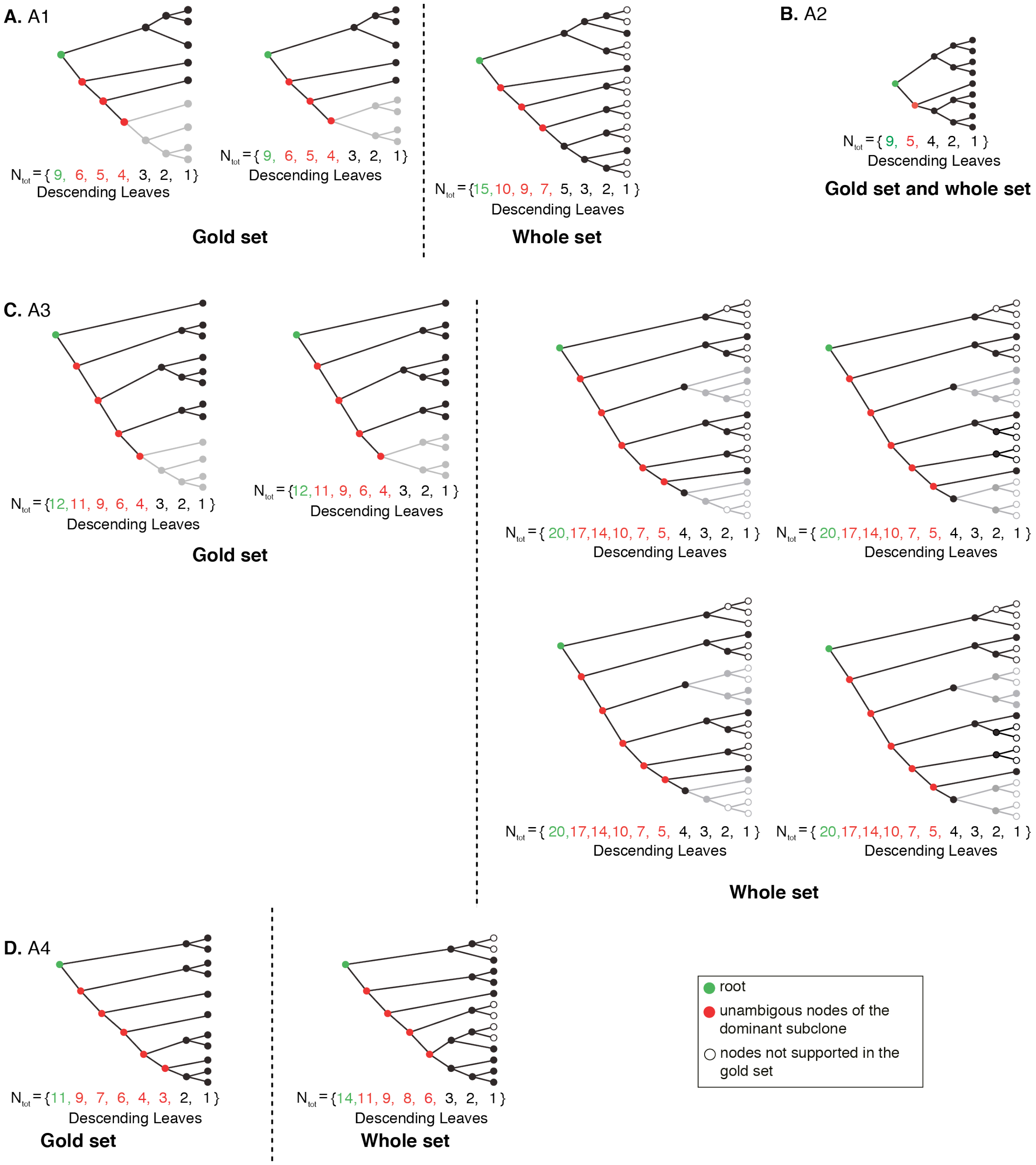

For each sample, all possible trees that fit with the combination of nodes are shown for the gold and for the whole sets of mutations. Combinations of external nodes (N_{tot}) were derived by dividing the frequency of each cluster by the frequency of the lowest cluster (C_{min}). In the gold set of A1 and A3, two trees are available because the combination {4, 3, 2, 1} results in two different subtrees (grey). For a similar reason, in the whole set of A3, four trees are

compatible because the combination {4, 3, 2, 1} appears twice in the tree reconstruction. It should be noted that all these trees share the same core structure and only differ in the topology of minor branches. Although none of these possibilities can be excluded, the trees that maximize the number of internal nodes in each branch of the tree (Figure 5B) should be preferred because they make no *a priori* assumptions on proliferation dynamics. In A2 and A4 only one tree is available for both the gold and the whole sets of mutations.

Figure S6. Robustness assessment of tree reconstruction.

A

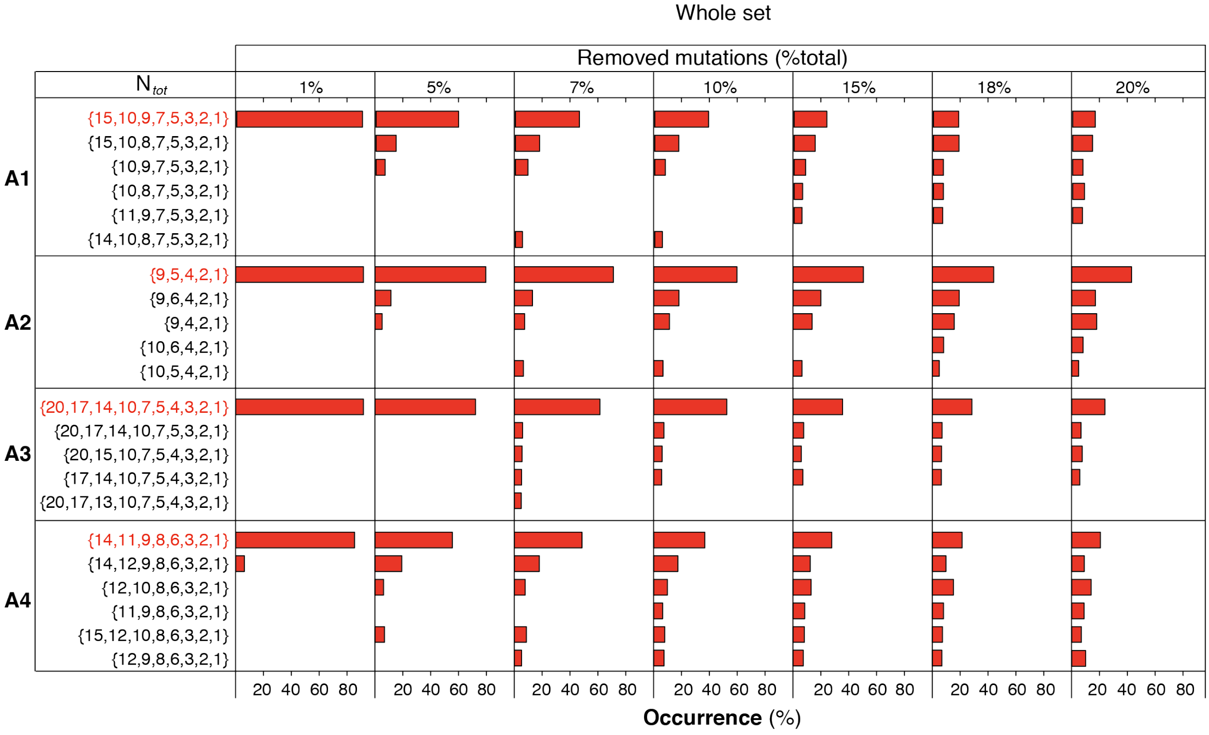

B

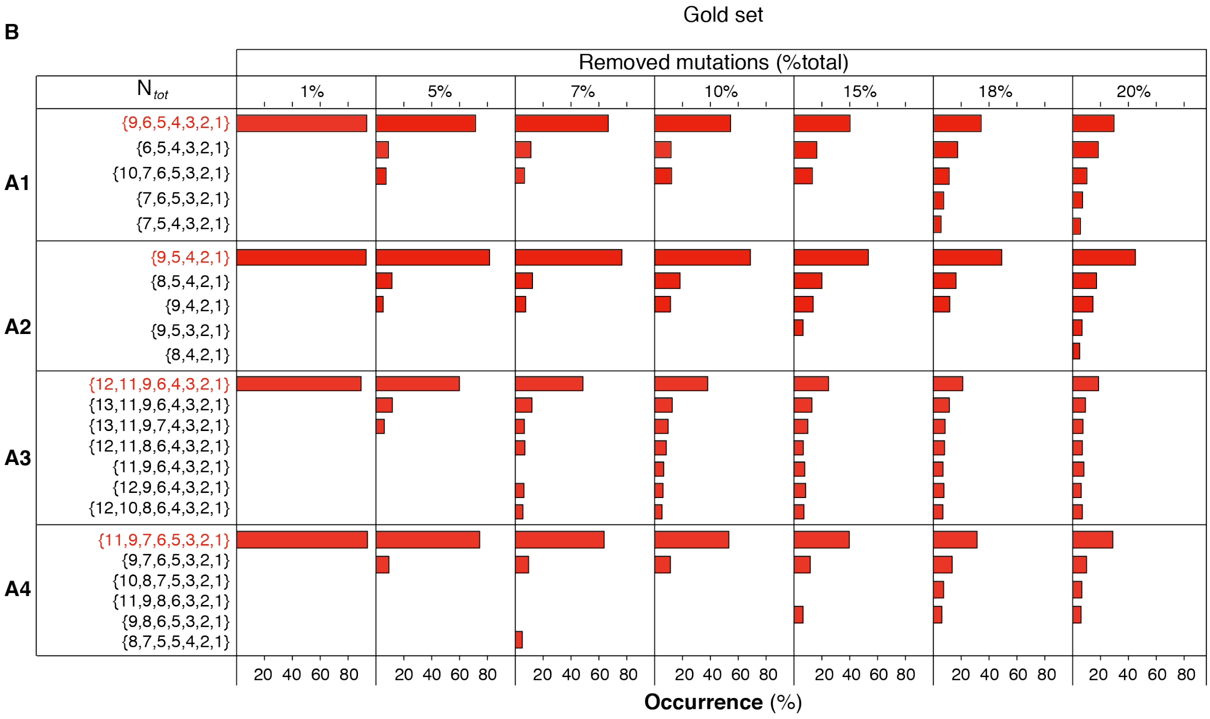

For each sample, shown are the results of a simulation study in which a variable fraction of the total mutations (from 1% to 20%) were randomly removed from the whole (A) and from

the gold (B) sets of mutations for 1000 times. At each iteration, the combination of nodes was re-assessed. For all four samples, the most frequent combination of nodes is always the observed one (red). It should be noted that the second most frequent combination only differs for one node from the observed one.
